# Tungiasis en el área urbana de Popayán, Colombia: reporte de caso

**DOI:** 10.7705/biomedica.5851

**Published:** 2021-05-31

**Authors:** Alicia Ortega-Narváez, Luis Reinel Vásquez-Arteaga, Olga Cujar-Otero, Jehyson Madroñero-Daza, Ginna Cabra-Bautista

**Affiliations:** 1 Grupo de Investigación en Lactancia Materna y Alimentación Complementaria, Departamento de Pediatría, Facultad de Ciencias de la Salud, Universidad del Cauca, Popayán, Colombia Universidad del Cauca Departamento de Pediatría Facultad de Ciencias de la Salud Universidad del Cauca Popayán Colombia; 2 Centro de Estudios en Microbiología y Parasitología - CEMPA, Departamento de Medicina Interna, Facultad de Ciencias de la Salud, Universidad del Cauca, Popayán, Colombia Universidad del Cauca Departamento de Medicina Interna Facultad de Ciencias de la Salud Universidad del Cauca Popayán Colombia; 3 Departamento de Pediatría, Hospital Susana López de Valencia, Universidad del Cauca, Popayán, Colombia Universidad del Cauca Departamento de Pediatría Universidad del Cauca Popayán Colombia; 4 Secretaría de Salud Municipal, Popayán, Colombia Secretaría de Salud Municipal Popayán Colombia

**Keywords:** Tunga, tungiasis, parásitos, Colombia, Tunga, tungiasis, parasites, Colombia

## Abstract

La tungiasis es una ectoparasitosis endémica en Latinoamérica y está asociada a factores de riesgo como la ruralidad, la pobreza y la convivencia con animales. Popayán, una ciudad al suroccidente de Colombia, fue históricamente afectada por la tungiasis, tanto así que a sus habitantes los apodan "patojos" debido a la forma de caminar de sus habitantes infestados por la pulga. Hoy la enfermedad se creía eliminada. Se presenta el caso de un niño de 12 años procedente del área urbana de Popayán, que consultó por lesiones papulares de bordes circulares, centro negruzco y halo hiperqueratósico en ambos pies, de un mes de evolución. Por los hallazgos clínicos se sospechó tungiasis y se le administró ivermectina. Las lesiones se removieron quirúrgicamente y se enviaron para análisis parasitológico, el cual confirmó la presencia de *Tunga penetrans.* La evolución del paciente fue satisfactoria. La Secretaría de Salud Municipal de Popayán inspeccionó el domicilio del paciente y encontró perros migrantes del Pacífico colombiano en sus alrededores, algunos con lesiones sospechosas de tungiasis. Se documenta, así, el resurgimiento de esta enfermedad en el área urbana, probablemente debido a la migración de animales desde las zonas rurales. Es importante reconocer la existencia de la pulga en zonas rurales y urbanas, hacer el diagnóstico médico y reportar los casos a los entes de vigilancia. Estas acciones permitirán ofrecer un apropiado manejo y control sanitario de esta ectoparasitosis desatendida en humanos y animales.

## Abstract (en)

Tungiasis is an endemic ectoparasitosis in Latin America associated with risk factors such as rurality, poverty, and living with animals. In Popayán, a city in southwest Colombia, the disease was highly prevalent in the past, to such degree that its inhabitants were given the nickname of *patojo,* a word that describes the way of walking of people infested by the flea. However, it was believed eradicated at present.

We present the case of a 12-year-old boy living in the urban area of Popayán, who presented with a one-month history of papular lesions with circular edges, blackish center, and hyperkeratotic halo on both feet. Tungiasis was suspected based on the clinical findings and ivermectin was administered. The lesions were surgically removed and sent for parasitological analysis, which confirmed the presence of *Tunga penetrans.* The patient evolved satisfactorily. The *Secretaría de Salud Municipal de Popayán* carried out an inspection of the patient's home where migrant dogs from the Colombian Pacific were found in the surroundings, some with suspicious lesions of tungiasis.

We document here the resurgence of this pathology in urban areas, probably secondary to the migration of animals from rural areas. It is important to recognize the existence of the flea in rural and urban areas, make a medical diagnosis, and report cases to the surveillance entities to enable appropriate management and sanitary control of this neglected ectoparasitosis in humans and animals.

La tungiasis es una ectoparasitosis desatendida que afecta la piel y es producida por la hembra de *Tunga penetrans.* Esta pulga también se conoce como "nigua" (Argentina, Ecuador, Venezuela, Colombia y el Caribe), "pique" (Argentina, Chile, Uruguay y Paraguay), "chica" (Colombia y Venezuela), "kuti" (Bolivia) o "bicho do pé" (Brasil) ^1^^-^^7^. Es una enfermedad endémica en el África subsahariana, el Caribe y América Latina, donde tradicionalmente se ha asociado a poblaciones que viven en zonas rurales en condiciones marginales y precarias. En las comunidades endémicas, la prevalencia puede llegar hasta el 30 % en la población general y el 85 % en los niños ^3^^,^^8^^,^^9^.

La tungiasis era común en Colombia. No obstante, su incidencia disminuyó gracias a factores como la aplicación de insecticidas, las mejoras en las viviendas y el uso de calzado ^2^. Hay tres publicaciones de casos de tungiasis en el país que describen pacientes de regiones rurales o selváticas o en condiciones de precariedad; dos corresponden a reportes de caso de pacientes provenientes de áreas rurales ^10^^,^^11^ y la otra es una serie de casos de indígenas de la región amazónica ^12^.

Popayán es una ciudad del suroccidente de Colombia que fue históricamente muy afectada por la tungiasis, pero ante la ausencia de casos reportados, actualmente la enfermedad se creía eliminada en el área urbana.

Se presenta el caso de un niño de 12 años procedente del área urbana de Popayán, con lo cual se documenta la persistencia de la enfermedad en la región, no solo en condiciones de ruralidad y pobreza, sino también en áreas urbanas con adecuadas condiciones de higiene.

## Caso clínico

Se trata de un niño de 12 años, originario y procedente de Popayán, que consultó por presentar lesiones cutáneas no pruriginosas ni supurativas en ambos pies, de un mes de evolución. El niño tenía antecedentes de asma y rinitis alérgica y su esquema de inmunizaciones estaba completo. El paciente residía en una casa del área urbana de Popayán, construida con cemento y piso de baldosa, convivía con sus padres, un hermano y dos perros; ninguno presentaba lesiones similares. En la región posterior de la casa, había un patio de tierra con algunos cerdos con los que el paciente no había tenido contacto. La familia tenía buenas prácticas de higiene, usaban calzado dentro de la casa la mayor parte del tiempo, contaban con todos los servicios públicos y un adecuado acceso a la atención en salud. No habían realizado viajes a áreas rurales recientemente, pero la madre refirió haber presentado tungiasis diez años atrás.

**Figuras 1 y 2:**
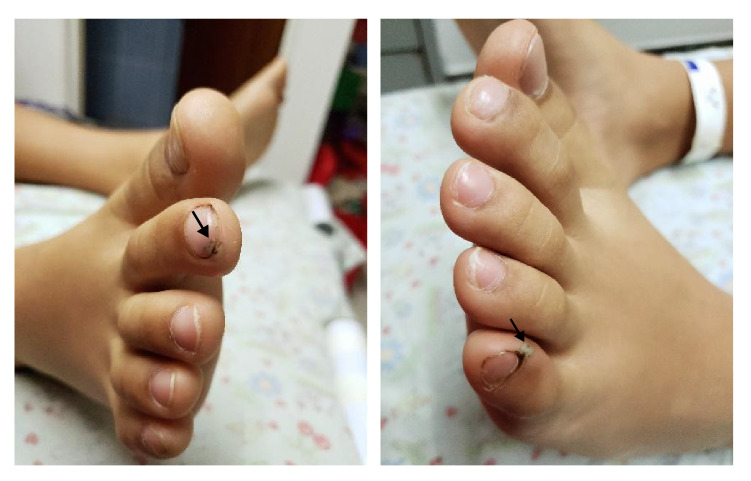
Lesión típica de tungiasis en estadio 3a. Se observa una pápula translúcida con borde bien definido y circular, y en el centro, un área puntiforme color negro y halo hiperqueratósico.

**Figura 3:**
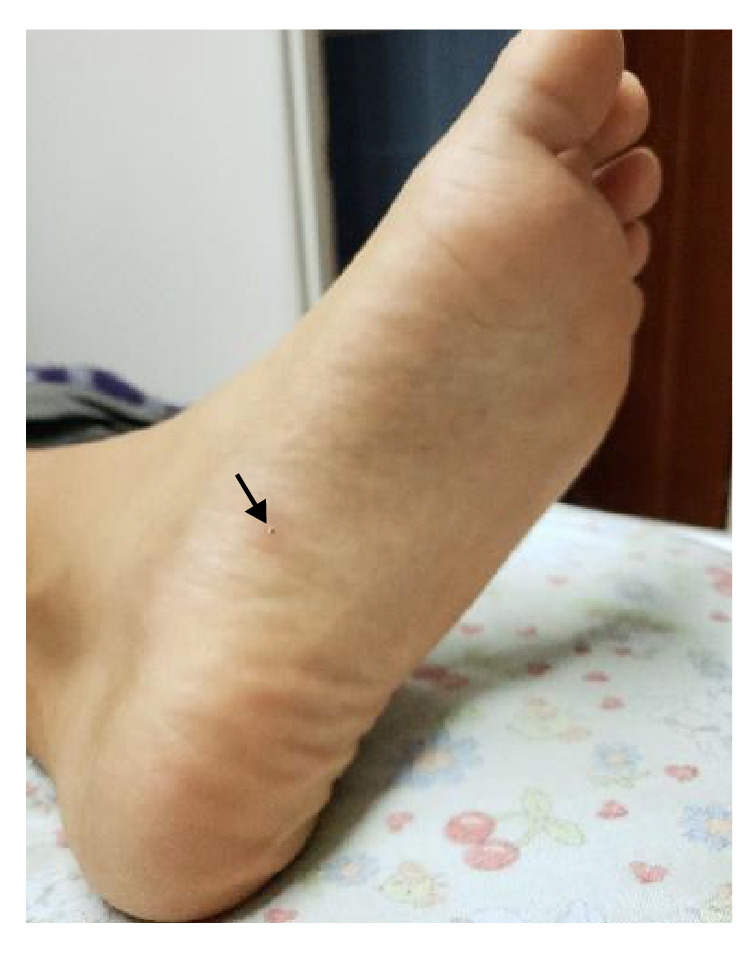
Lesión típica de tungiasis en estadio 5. Se observa una pápula circular difusa, desecada, con punto negro central cubierto de costra

En el examen físico se evidenciaron tres lesiones papulares con un halo blanquecino y un punto negro central: una en el borde ungular del segundo dedo del pie derecho (figura 1), otra en el borde ungular del quinto dedo del pie izquierdo (figura 2) y otra en la planta del pie izquierdo (figura 3). No había dolor a la palpación, ni sangrado o supuración.

**Figuras 4 y 5:**
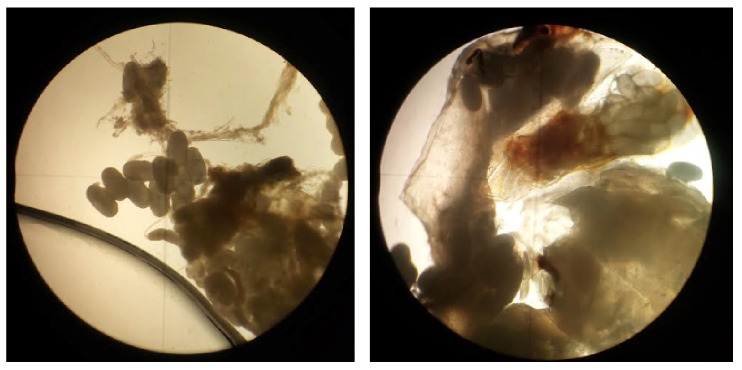
Restos de *Tunga penetrans.* Se observan el vientre y la cloaca del parásito, con presencia de huevos en el interior y exterior. Sin coloración, 4X.

Con base en las características clínicas, se diagnosticó tungiasis, se le administró una dosis única de ivermectina de 200 μg/kg y se le hizo la extracción quirúrgica de las lesiones, las cuales se enviaron a estudio. El análisis parasitológico confirmó la presencia de restos de hembras adultas de *T.penetrans,* con huevos en su interior (figuras 4 y 5).

El manejo del caso se complementó con un estudio de campo adelantado por el servicio de sanidad ambiental de la Secretaría de Salud Municipal de Popayán, en el cual se determinó que se trataba del primer caso confirmado de tungiasis en la historia de Popayán. Se visitó el domicilio del paciente y se encontró que había cerdos y perros en buen estado de salud y sin lesiones de tungiasis. En los alrededores del domicilio, se encontraron granjas porcícolas y sobrepoblación de perros (más de uno por cada 10 habitantes), algunos de ellos procedentes de zonas rurales del Pacífico colombiano.

Los perros evaluados presentaban lesiones eritematosas y pruriginosas en las almohadillas interdigitales anteriores, sugestivas de *T. penetrans* por su localización anatómica, aunque esto no se confirmó mediante el diagnóstico parasitológico.

Al mes de la consulta inicial, el paciente fue valorado en un control y se evidenció la resolución total del cuadro, descartándose la presencia de nuevas lesiones. Se obtuvo el consentimiento informado por escrito de la madre del paciente para la publicación de este informe del caso y de las imágenes.

## Discusión

Se trata del primer reporte primer reporte que confirma clínica y parasitológicamente la existencia de tungiasis en Popayán, una región tradicionalmente afectada, en la que la presencia de la enfermedad se había informado únicamente en documentos de historia y en su tradición oral ^4^^,^^6^^,^^7^. El sobrenombre de las gentes de Popayán es "patojo", término que alude a personas con los pies torcidos o cojos, cuya forma de caminar imita la de los patos. *Tunga penetrans,* más conocida en la región como "nigua" era muy frecuente en la ciudad y al introducirse en los pies impedía que los afectados caminaran normalmente, lo que dio origen al gentilicio ^3^^,^^4^^,^^6^^,^^7^.

El filósofo y escritor José Néstor Valencia Zuluaga relataba en su artículo "Nuestro pasado con las niguas" que, en su tesis de 1937, el médico francés Pierre Crouchet recogía cómo había constatado más de dos mil casos de tungiasis en Popayán, proponiendo que las niguas estaban relacionadas con la inteligencia de los "patojos", información cuya fuente no pudo encontrarse ^4^.

La historia también cuenta que "Don Quijote de la Mancha" se habría escrito gracias a las "niguas". Se dice que Cervantes se enfermó de tungiasis tras convivir con un amigo que llegó de Popayán infestado de pulgas; estas le habrían ocasionado a Cervantes un estado febril tan intenso que lo hizo imaginar las locas aventuras de su personaje ^4^.

**Figura 6:**
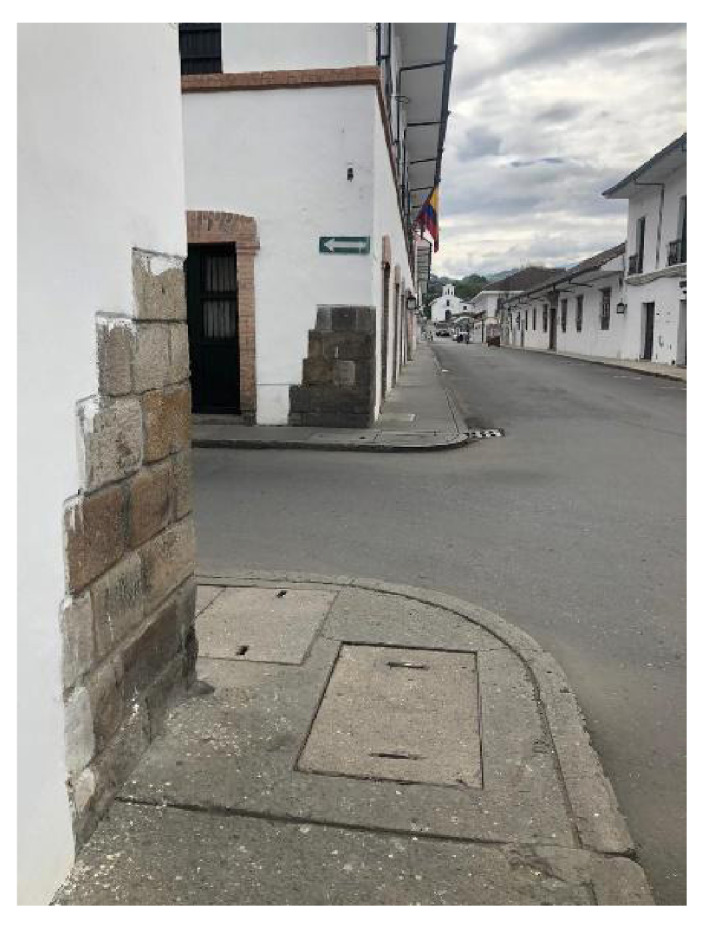
Piedras marfileñas en las esquinas de Popayán que servían a sus habitantes para rascarse y calmar el prurito producido por la tungiasis

La tungiasis influyó sobre la arquitectura y la cultura de esta ciudad colombiana conocida como la "Ciudad Blanca" Todas las paredes de su centro histórico son blancas debido a que en la época en que las niguas abundaban se pintaron las fachadas con cal para erradicarlas. Además, eran frecuentes las piedras marfileñas incrustadas en las esquinas de las casas del centro de la ciudad, las cuales servían a sus habitantes para rascarse los pies (figura 6), tal como lo señala Horacio Dorado: "Era costumbre rascarse en las piedras de las calles" ^7^.

Con el desarrollo de la ciudad, la pavimentación de carreteras, los insecticidas y el uso de calzado, la carga de tungiasis fue disminuyendo ^4^^,^^6^. El historiador Octavio Hernández Jiménez escribía: 'A finales del siglo XX se habían habilitado los subterráneos con nuevos cuartos o viviendas, se habían pavimentado y ya no había terneros, vacas, perros o gallinas para guardar, se acabaron del todo las niguas" ^6^. Según estos relatos. la tungiasis parecía ser una enfermedad ya extinta en la ciudad de los patojos. Sin embargo, el caso descrito nos muestra lo contrario.

### Epidemiología

La tungiasis es una ectoparasitosis endémica en Latinoamérica; no obstante, en Colombia no hay datos sobre su frecuencia a nivel nacional. Solo se encontró un estudio en una población indígena amazónica que reportó una incidencia de tungiasis de 3 a 8 casos por cada 1.000 indígenas y compromiso del 62 % de los perros ^2^.

La prevalencia por edad sigue una distribución en forma de S, con un pico en niños entre 5 y 14 años y otro en ancianos ^8^. El paciente reportado se encontraba en el rango de edad considerado como pico para la tungiasis.

Los factores de riesgo típicos de la enfermedad incluyen la residencia en áreas rurales o costeras, la pobreza, el habitar en casas con piso de arena o tierra o caminar sobre suelos de tierra o arena, el no usar calzado, la mala higiene, el abandono social, y la falta de educación y de atención en salud ^2^^,^^13^. Contrario a ello, el paciente que reportamos vivía en una zona urbana en la que no se evidenciaban estos factores.

La tungiasis afecta un amplio espectro de animales, tales como perros, gatos, cerdos, vacas y ratas, por lo que los patrones de cohabitación entre animales y humanos tienen un papel fundamental en la dinámica local de transmisión de la enfermedad. Feldmeier, *et al.,* plantean el control de los criaderos y reservorios animales como estrategia para combatirla ^8^. En ciudades intermedias como Popayán, es frecuente encontrar patrones de convivencia con ganado bovino y porcino y con otros animales, factor que estuvo presente en el caso reportado, aunque en el domicilio del niño los animales se encontraban sanos. De todas maneras, dicha convivencia podría propiciar la transmisión de la enfermedad en este tipo de ciudades.

En algunos estudios en África se ha evidenciado que la tungiasis ha empezado a extenderse más allá de las áreas rurales como consecuencia de la construcción de carreteras y el aumento de la movilidad humana, lo que ha permitido que *T. penetran*s viaje largas distancias desde el área rural hasta las urbes; además, la movilización de los reservorios animales ha sido clave para esto ^8^. Este mecanismo podría explicar el probable resurgimiento actual de la tungiasis en el área urbana de Popayán, ya que el análisis de campo evidenció la migración de perros del área rural hacia la zona urbana, los cuales podrían actuar como reservorios de *T. penetrans* y transmitir la enfermedad a animales y humanos de la ciudad.

### Clínica

Las manifestaciones de la tungiasis están asociadas con el ciclo de vida de la pulga hembra dentro del huésped. Cerca del 99 % de las lesiones ocurre en los pies debido a que la pulga salta un máximo de 20 cm y los sitios más afectados son las regiones periungulares, los pliegues interdigitales y las plantas ^9^^,^^13^^,^^14^. Las lesiones incluyen pápulas, nódulos o placas únicas o múltiples, grisáceas o marrones, caracterizadas por una triada clínica clásica que comprende un punto negro central, correspondiente a la cloaca, un halo translúcido alrededor, que es el abdomen lleno de huevos, y un área de hiperqueratosis periférica como reacción al cuerpo extraño en la piel circundante ^14^^-^^16^.

La reacción inflamatoria que se desarrolla alrededor de la pulga enterrada es la causante de la sintomatología de la tungiasis, caracterizada por eritema, edema, prurito, dolor y calor locales, la cual, a medida que progresa, aumenta la frecuencia de la sobreinfección bacteriana ^8^^,^^13^. La llamada clasificación de Fortaleza, propuesta en el 2013, describe la historia natural de la tungiasis humana y la divide en cinco etapas ^14^. Según esta clasificación, en el presente caso las lesiones se encontraban en estadio 3a (figuras 1 y 2) y 5 (figura 3).

### Complicaciones

La tungiasis es una enfermedad que puede generar gran morbilidad, aguda y crónica. Algunas de las complicaciones más frecuentes son abscesos, úlceras profundas, flemones, linfangitis, osteomielitis, neuritis ascendente, fascitis, gangrena y sepsis, y su asociación con micosis profundas y tétanos. Puede llevar a la necrosis de tejidos blandos, de ligamentos o de huesos, y provocar la autoamputación de los dedos con la consecuente deformidad y alteración de la marcha ^8^^,^^13^^,^^14^^,^^17^.

La tungiasis grave es común en áreas con gran frecuencia de reinfección, en donde las condiciones de higiene son precarias, y es más frecuente en niños y ancianos ^8^^,^^9^. Es una enfermedad que puede generar discapacidad y pérdida de la calidad de vida ^8^^,^^9^^,^^14^. Afortunadamente, el paciente reportado cursó con una enfermedad leve y no tuvo ninguna complicación.

### Diagnóstico

El diagnóstico se hace con base en los hallazgos clínicos a partir de la morfología y la topología de las lesiones, teniendo en cuenta los posibles factores de riesgo y la historia natural de la enfermedad ^14^. Mediante dermatoscopia, se puede visualizar la triada clínica clásica característica, el exoesqueleto y, en ocasiones, los huevos de la pulga ^16^.

El diagnóstico histopatológico no está indicado de forma rutinaria. Sin embargo, se puede hacer biopsia de las lesiones, la cual puede evidenciar en el tejido áreas de hiperqueratosis, paraqueratosis, acantosis y espongiosis; a veces, es posible visualizar la morfología de la pulga y sus huevos ^9^^,^^14^^,^^18^. En este paciente se logró el diagnóstico parasitológico y se observaron *T. penetrans* adultas con huevos en su interior.

Entre los diagnósticos diferenciales se encuentran verrugas, infecciones y abscesos piógenos, micosis profundas, paroniquia aguda, reacción a cuerpo extraño, escabiosis, miasis, larva *migrans,* melanoma, quiste dermoide y mordedura o picadura de otros artrópodos ^2^^,^^18^.

### Tratamiento

El tratamiento definitivo es la extracción quirúrgica de las lesiones, procedimiento que debe llevarse a cabo bajo condiciones de asepsia y tan pronto se haga el diagnóstico, por el riesgo de sobreinfección bacteriana; además, deben retirarse todas las lesiones completamente, pues la persistencia de cualquier resto provoca una reacción inflamatoria intensa. En las áreas rurales, es común el uso de métodos de extracción no estériles, con instrumentos como agujas y espinas ^9^^,^^14^^,^^18^.

Tras la extracción, se recomienda aplicar un antibiótico tópico para evitar la sobreinfección bacteriana ^9^^,^^18^. Además, existe riesgo de infección por *Clostridium tetani* debido a que *T. penetrans* puede recoger esporas de estas bacterias, que también pueden adquirirse en extracciones que no se hacen con la debida asepsia, por lo cual en algunas publicaciones se recomienda aplicar profilaxis para tétanos a aquellos pacientes infectados que no hayan sido vacunados ^2^^,^^8^^,^^9^^,^^18^. En el caso reportado, se empleó un método de extracción estéril y el niño tenía su esquema completo de vacunación, por lo que no fue necesaria la aplicación del toxoide tetánico.

Ningún medicamento ha demostrado ser efectivo en el tratamiento de la tungiasis ^9^^,^^13^. La ivermectina se ha empleado ampliamente con base en la observación anecdótica de su efectividad; sin embargo, en un estudio clínico aleatorizado, se documentó que el uso de ivermectina no era más efectivo que el placebo ^19^. En estudios recientes se ha evaluado la efectividad del tratamiento tópico con una dimeticona conocida como NYDA™, con resultados esperanzadores. La dimeticona se aplica sobre el cono abdominal trasero de la pulga que sobresale por encima de la piel, ya que allí terminan y empiezan varios sistemas vitales del parásito, con lo que se ataca sus sistemas respiratorio, digestivo y genital ^3^. La tungiasis es una enfermedad desatendida y falta evidencia sobre este tipo de tratamiento. En el paciente reportado, las lesiones mejoraron tras la extracción total, aunque también recibió una dosis de ivermectina.

### Prevención

La prevención basada en la mejora de las condiciones de vida y el control de los factores de riesgo modificables son los únicos medios disponibles para combatir la transmisión y la infestación ^8^^,^^9^. En un estudio en la Amazonia colombiana, se demostró que el cambio de piso de tierra por piso sólido, la humectación y rociado con piretroides, más un tratamiento simultáneo y oportuno de humanos y reservorios, han dado resultados favorables en la población intervenida ^2^. El uso de calzado cerrado es una buena medida en aquellas poblaciones en donde no es frecuente usarlo, así como la autoexploración diaria para detectar lesiones incipientes; estas medidas son factores de protección que disminuyen la presencia de tungiasis ^20^. En otros estudios se ha evaluado la efectividad del Zanzarin™, un repelente de origen vegetal que contiene aceite de coco, extracto de jojoba y *Aloe vera,* el cual reduce la tasa de ataque, la intensidad de la infestación y la morbilidad asociadas con la tungiasis ^21^^,^^22^.

## Conclusión

Se presenta el caso de un paciente con tungiasis, procedente de un área urbana, lo que evidencia el resurgimiento de la enfermedad en la ciudad, probablemente por la migración de animales desde zonas rurales.

Es posible que exista un subregistro de esta enfermedad y de su reporte a los entes de vigilancia, debido al desconocimiento que hay sobre ella y la falta de sospecha diagnóstica. Es necesario implementar acciones para un manejo adecuado de esta condición tanto en humanos como en animales, así como brindar educación en la prevención, hacer búsqueda activa de los focos de tungiasis en áreas urbanas y llevar a cabo el control sanitario. En este reporte se demuestra el resurgimiento de la tungiasis en la ciudad de los "patojos" y, probablemente, en otras urbes.
